# The Power of the College of Nursing

**Published:** 1918-09-14

**Authors:** 


### THE NURSE'S VOICE.

### THE POWER OF THE COLLEGE OF NURSING.

Probably not one nurse in a thousand read about or took the slightest interest in the Metropolitan Police Strike which took place recently, and which ended with the minimum of delay. Nevertheless one particular feature of the trouble had a peculiar interest for nurses. The police are a disciplined Force, and one of their regulations is that they must not memorialise their authorities. If a constable suffers from any grievance, financial or otherwise, he may complain, if it so pleases him, but he commits a breach of discipline if he goes round amongst his comrades and asks them to join him in a "round robin" to their authorities, although the grievance may be common to the whole establishment. On the occasion in question the whole Metropolitan Force felt that they suffered from the most trying of all grievances, inadequate remuneration, and they secretly combined to express themselves by striking.

### Inarticulate by Order.

I am not concerned with the question of police pay, and I am quite unable to offer any opinion as to whether their grievance was real or imaginary. But I am quite sure that it would never have taken place but for the fact that they were, by regulation, inarticulate. The Prime Minister at much inconvenience had to leave his pressing duties in connection with the war, and it is worthy of note that the course which he adopted was to remedy the financial trouble, and at the same time to promise to provide a machine by which in the future the police will be able, without suffering in any way, to approach their authorities and speak their mind. It was a masterful stroke of the Premier's. All the obsolete traditions of discipline were swept away as with a besom, and the men hurried back to their patrols with cheerful minds.

### Why the Nurse Remains Silent.

As I read this in the *Times* I immediately thought of the British nurses, and of the scores of confessions which I have received from hospitals in the Metropolis and in the provinces that fear, and nothing else, prevents them from saying what they think. The nurse, of course, is not forbidden to articulate, nor does she belong to what is known as a disciplined body. Nevertheless she realises that silence is effectually imposed upon her, and that to appear in the guise of an agitator is to court punishment, if not loss of office, and in every way to ruin her prospects. And it is here that the College of Nursing has an opportunity of coming to her rescue, and of establishing itself as the go-between in the case. The College is the Nurses' Union. Before its arrival there were scores of smaller unions, all of which failed to make good, and for the obvious reason that not one of them had the courage or the wit to face the economic problem, which, after all, is the only problem that presents any difficulty.

### The Votes of Ten Thousand Nurses.

The College is an institution with enormous potentialities, and I would urge upon the attention of the Council the absolute necessity for economic reform, upon which, as I endeavoured to show last week, educational reform really depends. I said once before that it ought to be a Nurses' Parliament, and when elections take place for representation on the Council I should like to see some competition, and a statement of policy by those offering themselves as candidates. It would be impertinent for me to dogmatise as to what nurses as a body think, and it would always be open to ladies like "Matron," who recently wrote to The Hospital, to suggest that I am the victim of my imagination. Let us suppose that there were an election to-morrow, and that the eight-hour question were put to the vote. "Matron" considers if her nurses were granted this privilege or relief that they might get into mischief, and that such liberty would be bad for the profession. On this question I should very much like to see the recorded votes of the ten thousand nurses who have become members of the College. I have a suspicion that if the wishes of the profession were consulted, the poll might surprise many who hold, that this question would mean mischief.

### Do Matrons Cease to be Nurses?

Now I want to ask the College Executive a plain question—Are they trying to interpret the wishes of the inarticulate nurse, and, having interpreted them, are they determined to press their claims for reform on the attention of hospital boards? The success or failure of the College as a great British Imperial institution depends on the answer to this question. An out-and-out believer in the College as a potential engine for good, I hold that sooner or later every member of the Executive must realise that the problem of economic reform is of paramount importance, and that it must be faced with fearless determination. A common objection to the Executive is that it includes laymen. My objection is that there is a preponderance of biassed professional representation. There are too many matrons, and I am not aware that any of them have ever put forward a scheme for reform based on the wishes and aspirations of the rank and file. I do not suggest that their views diverge from those of the rank and file. They may be identical; but there is no evidence thereof. One of the features of Parliament is that every man who occupies a seat publishes his politics for the consideration of his constituency prior to his election, and one of the bulwarks of British freedom is that the voice of the people is entitled to a hearing. With ten thousand members the College of Nursing possesses the power to speak with authority. It can command a hearing in every hospital board in the kingdom.

### The Nation and the Nurse.

Reform is on the wing. If it is slow in arriving, the profession must deteriorate. The best candidates will seek employment in other fields, and recruits to nurse the nation's sick poor will come from a lower stratum. Women of talent and breeding in this the hour of their emancipation will not consent to an eleven-hour day, and meals served as occasion demands in the sick wards. I decline to believe that the British nation demands such service. And to come back to the police, it is abundantly evident that the British nation did not approve of their conditions of service, a fact which the Prime Minister demonstrated with a stroke of his pen.

IERNE.

☛ For letter "Matrons and Nurse Agitators," see page 525.

